# Molecular analysis of iduronate -2- sulfatase gene in Tunisian patients with mucopolysaccharidosis type II

**DOI:** 10.1186/1746-1596-6-42

**Published:** 2011-05-23

**Authors:** Latifa Chkioua, Souhir Khedhiri, Salima Ferchichi, Rémy Tcheng, Henda Chahed, Roseline Froissart, Christine Vianey-Saban, Sandrine Laradi, Abdelhedi Miled

**Affiliations:** 1 Biochemistry Laboratory Farhat Hached Hospital, 4000 Sousse, Tunisia; 2 Biology Molecular Laboratory University of Pharmacy, 5000 Monastir, Tunisia; 3 Hereditary service of metabolic diseases and neonatal screening. Center of biology and pathology. 69677 BRON CEDEX, France

## Abstract

Mucopolysaccharidosis type II (MPS II, Hunter syndrome) is X-linked recessive lysosomal storage disorder resulting from the defective activity of the enzyme iduronate-2-sulfatase (IDS). Hunter disease can vary from mild to severe, depending on the level of enzyme deficiency. We report the IDS mutation and polymorphisms causing the Hunter syndrome in patients from one family in Tunisia

**Patients and methods:**

A preliminary diagnosis was made by qualitative detection of urinary glycosaminoglycans of the suspected MPS II probands. The IDS mutation and polymorphisms were determined on these probands and their family members by amplifying and sequencing each of the exons and intron-exon junctions of IDS gene.

**Results:**

The studied probands were homoallelic for p.R88P mutation. In addition, three known polymorphisms/sequence variants: IVS3-16 (c.419-16 delT), T214M (c.641C > T), T146T (c.438 C > T), IVS5-87(c.709-87G > A) and one previously unknown: IVS7+38(c.1006+38T > C were identified in the MPS II patients. These are the first Tunisian MPS II patients to be genotyped.

**Conclusion:**

The identification of these mutation and polymorphisms and their genotype-phenotype correlation should facilitate prenatal diagnosis and counseling for MPS II in Tunisia, where a very high rate of consanguinity exists.

## Introduction

Mucopolysaccharidosis type II (MIM 309900) is X-linked recessive lysosomal storage disorder caused by the deficient of the enzyme iduronate 2-sulfatase (IDS, EC 3.1.6.13). This glycosidase is required for the hydrolysis of the 2-sulfate groups of the L-iduronate 2-sulfate units of dermatan sulfate and heparan sulfate [1]. Deficiency of this enzyme causes accumulation and higher urine excretion of these undegraded substrates in lysosomes leading to cell death and to the clinical manifestations of the disease. A wide spectrum of clinical severity is recognized in MPS II. Severely affected individuals usually die between 10 and 15 years of age, although some die much earlier. Individuals with mild forms of MPS II can have significant somatic disease, but reasonably normal life spans. The treatments for affected individuals were bone marrow [2] and enzyme replacement therapy [3]. A preliminary diagnosis was made by qualitative detection of urinary glycosaminoglycans of the suspected MPS II proband, then, confirmed by the demonstration of deficient iduronate 2 sulfatase activity in leukocytes, plasma or cultured cells. The IDS gene is situated on the telomere of the long arm of chromosome X at region Xq28 [4]. Two forms of IDS were identified from human placenta [5] and 2 major forms of iduronate sulfatase were purified with molecular masses of 42 kD and 14 kD [6]. The gene has 9 exons and produces a transcript of 1.4 that encodes a precursor protein of 550 amino acide [7]. More than 300 different mutations in the IDS gene had been reported in patients with Hunter syndrome [8] including gene rearrangements caused by recombination with the IDS2 pseudogene, deletions of certain exons or the entire IDS gene or small mutations including insertions, deletions and point mutations that created a novel splice site. A pseudogene IDS2 also exists 20 kb from the active IDS gene. This pseudogene shares homology to exon 2, intron 2, exon 3, intron 3 and intron 7 of the IDS gene [9].

Mucopolysaccharidosis type II has two major clinical phenotypes, ranging the severe form called MPS IIA to the mild form called MPS IIB:

The severe form (MPS IIA), the child is normal at birth, and symptoms appear gradually. The diagnosis is usually referred to between 2 and 4 years. The MPS IIA patient is characterized by progressive mental retardation, physical disability, severe airway obstruction, skeletal deformities, cardiomyopathy, and, in most patients, neurologic decline. Death usually occurs at age of 15 years in most cases.

The mild form (MPS IIB), the clinical symptom appears after 10 years of age. At this age, the MPS IIB patient had normal growth and development. He had mild dysostosis radiologically, coarse facial features, flexion contractures of the elbows and shoulder joints, moderate hepatosplenomegaly and a long life span with and a long life span with minimally impaired neurological. A lack of opacification of the cornea was noted in the MPS II as opposed to the MPS I [10].

This inherited disease may constitute a relatively more important social and economic concern in Tunisia because of the prevalence of first-cousin marriages [11]. Thus, the identification of the IDS mutations causing MPS II in Tunisia is important for prenatal diagnosis in affected families. Here, we report the IDS mutation and polymorphisms causing the Hunter syndrome in patients from one family in Tunisia.

## Patients and methods

### Patients

One family from Tunisia with MPS II was investigated. The MPS II patients were diagnosed in paediatric department of center Tunisia.

**Figure 1:**
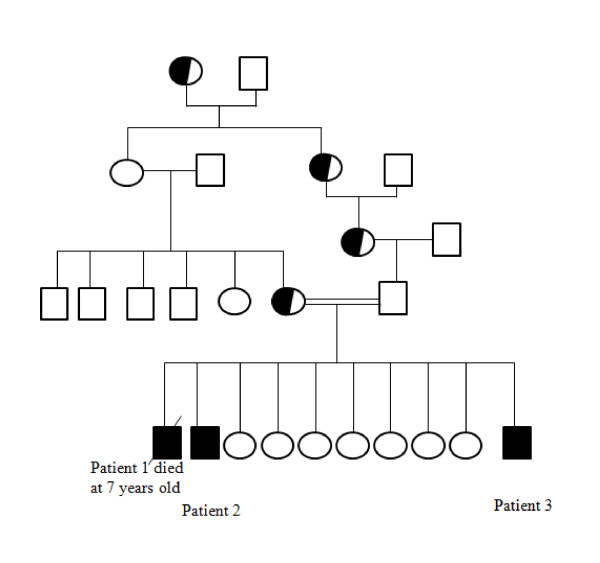
**Pedigree of studied family**. Square and circles indicate male and female members, respectively. Shaded symbols indicate affected individuals.

This family gave birth for 3 affected children (Figure 1). The first patient was died before our investigation at on age of seven years. The other two boys: the patient 2 was diagnosed at the age of three years when he was operated for inguinal hernia. Coarse facial feature including macrocrania, macroglossia and small teeth and poorly implemented were noted at an age of eighteen months. He had a short stature (-4DS), hepatosplenomegaly, skeletal disease and he had developed progressive mental retardation. Then, he deceased as a result of cardiorespiratory disease. The patient 3 was developed similar coarse facial features at the age of eighteen months. He showed severe developmental delay, hydrocephaly, deafness and inguinal hernia.

The MPS II patients were offspring of consanguineous marriage between first cousins (Figure 1). Blood samples were collected from the patients, their parents, and unaffected siblings and relatives. The clinical diagnosis was confirmed enzymatically (Table 1). This study was approved by the Ethnics Committees of the Tunisian Hospital and the parents gave full informed consent.

**Table 1:** the clinical features of MPS II patients and their leukocyte IDS activity

Features	Family 1	
	**Patient 1**	**Patient 2**

MPS II genotype	p.R88P	p.R88P

IDS activity nmol/h/mg protein	2	5

Prenatal diagnosis	1^st ^cousins	1^st ^cousins

Age at diagnosis (year/month)	3 years	3 years

Age of onset (year/month)	18 months	18 months

Sex	Male	Male

Growth retardation	Marked	Marked

Macroglossia	Marked	Marked

Macrocarinia	Marked	Marked

Hepatosplenomegaly	Marked	Marked

skeletal deformities	severe	severe

Other features	teeth small and poorly located deafness	teeth small and poorly located deafness

Mental retardation	severe	severe

IDS activity nmol/h/mg protein	2	5

### IDS activity and Mutation analysis

IDS activity was determined in sonicated leukocytes pellets as described using the artificial substrate 4-methylumbelliferyl-alpha-iduronide-2-sulfate [12]. For IDS mutation analysis, genomic DNA was extracted from peripheral blood leukocytes by the phenol-chloroform procedure. Each of the 9 exon and intron-exon boundaries of IDS gene were amplified from genomic DNA from the patient and her parents as described previously [13]. The typical PCR reaction was carried out in a 50 μl total volume containing 100 ng of genomic DNA, 0.2 mmol/L dNTPs, 0.4 μM of each primer, 1.5 mmol/L MgCl_2_, 10% (V/V) DMSO and 0.15 μl (0.75 units) of Taq polymerase (Bromega).

## Results

*Clinical findings*: the clinical features of MPS II patients and their leukocyte IDS activity are presented in Table 1.

### IDS mutation analysis

The patients from southern Tunisia were homozygous for a G to C transversion in exon 3 predicting an arginine to a proline missense mutation p.R88P. In addition, five sequence variants, including four previously reported polymorphisms, were detected. The unreported polymorphisms was IVS7+38 (c.1006+38T > C). The previously described polymorphisms were IVS3-16 (c.419-16 delT), T146T (c.438 C > T), T214M (c.641C > T), IVS5-87(c.709-87G > A) (Table 2).

**Table 2:** Mutation and polymorphisms in MPS II patient

Mutation	Nucleotide change	Position cDNA	Codon	Exon/Intron	References	polymorphisms	Exon/intron	References
						IVS3-16 c.419-16 delT	4	
						
						T146T c.438 C > T	4	[31]
						
p.R88P	G > C	263	88	3	[28]	T214M c.641 C > T	5	
						
						IVS5-87 c.709-87 G > A	6	
						
						IVS7+38 c.1006+38 T > C	7	This report

## Discussion

Estimates of the incidence of MPS II in European and Anglo-Saxon countries range from 1:55 000 to 1:160 250 male newborns [14-20]. It is the most common type of MPS in Taiwan and northern Asia [21,22].

In Tunisia, the global incidence of all the MPS is 2.3/100.000 lives births [23]. A prevalence of different types of mucopolysaccharidoses was evaluated as the MPS type I, III and IV [24] but that of type II is unknown due the ignorance of some clinical picture.

Furthermore, MPS II is expected to be found only in males, but some females have been reported [25,26].

The diagnosis of MPS II patients was made by demonstrating an increased amount of urinary heparan sulfate and dermatan sulfate, and very low residual IDS activity in peripheral blood leukocytes.

According to the HGMD database, to date, more than 300 different mutations in the IDS gene had been reported in patients with Hunter syndrome (Human Gene Mutation Database; http://www.hgmd.org). The mutational spectrum associated with the IDS gene is quite heterogeneous and ranges from point mutations to large-scale gene conversion or deletion. The majority of mutations are missense and nonsense mutation, and approximately 15% of mutations are presumed to be caused by a gross deletion.

This study identified the first IDS mutation in Tunisian patients with the Hunter syndrome. These patients, whose were born to consanguineous parents, were homozygous for the previously described p.R88P missens mutation [27]. The p.R88P mutation resulted in the replacement of a basic arginine with a neutral proline. A non conservative substitution could be predicted to drastically change the orientation of the secondary structure in IDS protein. This mutation causes a sever instability or loss of IDS protein function leading to a severe disease.

**Figure 2:**
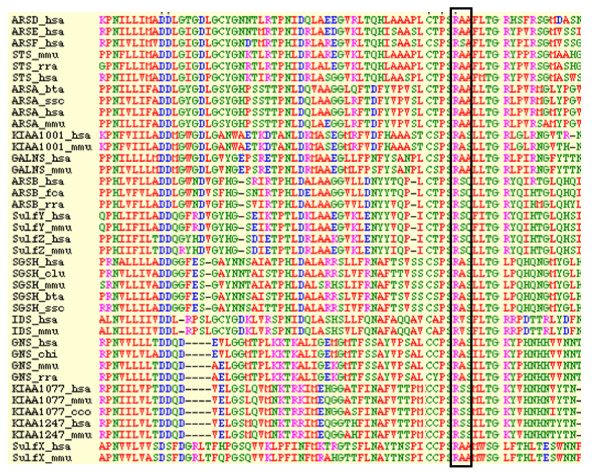
**Comparison of the homology of eukaryotic sulfatase**. Human Arginine 88 and homologous Arginine are indicated in pink and boxed.

**Figure 3:**
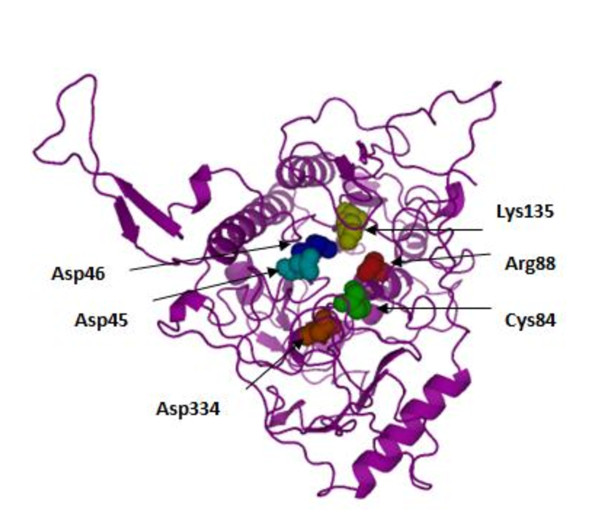
**Location of mutated residue (Arg88) in a tertiary structural model of IDS**. The active site centre, Cys84 residue, is shown as green spheres and the other active site residues are shown as different spheres colors.

Homology analyses revealed that arginine-88 is highly conserved among the human and eukaryotic sulfatases (Figure 2). The structure of human IDS was modeled by homology with crustal structure of human N-actyl-galactosamine-4-sulfatase (4S) and arysulfatase A (ASA) [28]. Therefore, residue 88 appears to be not essential for processing but important for IDS conformation [29]. R88 residue is found adjacent to the active site residue, C84, and is a part of the core of the major domain of IDS. The alteration by proline, a more bulky hydrophobic residue, can affect the stability of the major domain structure. The side-chain of R88 stretches in the opposite direction from the active site and arginine has a hydrophobic root in its side-chain. Thus, the substitution of R88 by a smaller hydrophobic residue, proline, could be tolerated (Figure 3).

In the present study, five IDS sequence variants (IVS3-16 (c.419-16 delT), T214M (c.641C > T), T146T (c.438 C > T), IVS5-87(c.709-87G > A). have been identified in Tunisian MPS II patients, including one novel polymorphism (IVS7+38 (c.1006+38T > C). Since the T146T substitution has frequently been described as a polymorphism site [30]. This modification was identified in homozygous state in patient with a severe phenotype [31] and also in patient with mild phenotype [13]. Our study showed that this modification was associated with a previously reported p.R88P mutation and others described polymorphisms and unreported polymorphism.

The effect of these polymorphisms on the expression of IDS is unknown. Further studies on large number of MPS II patients and normal population might help in finding out whether these variants are associated with MPS II or not. Moreover, the correlation between genotype and phenotype has remained unclear.

We note a considerable heterogeneity of MPS I [32] and MPS IVA [33] mutations in Tunisia; we also estimate that more mutations could be potentially present in the population.

The identification of a large battery of mutations in MPS patients including MPS I, MPS II and MPS IVA patients and the possibility of their early detection represent a cornerstone for the establishment of MPS prevention program in Tunisia based on prenatal diagnosis.

## Conclusion

In conclusion, this is the first report of IDS mutation in Tunisian patients with MPS II. We applied the following strategy: An accurate biochemical test is available for the diagnosis of Hunter disease consisting of the demonstration on deficient IDS activity in leucocytes. This test was performed before molecular analysis. Molecular identification of the mutation in individuals with a confirmed diagnosis can be used for carrier detection of the females in family at risque and prenatal diagnosis.

## Consent

Written informed consent was obtained from the patient for publication of this case report and accompanying images. A copy of the written consent is available for review by the Editor-in-Chief of this journal.

## List of abbreviations

MPS II: Mucopolysaccharidosis II; IDS: Iduronate -2-sulfatase; PCR: polymerase chain reaction; MPS I: mucopolysaccharidosis I; MPS IVA: mucopolysaccharidosis IVA.

## Competing interests

The authors declare that they have no competing interests.

## Authors' contributions

LC, SK and RT have done all the work (PCR, sequencing...) in the laboratory. SF, RF and HC analysis the results. CH VC, SL and AM has given final approval of the version to be published. All authors read and approved the final manuscript.
